# Supplementary material for: Clinical sequencing of soft tissue and bone sarcomas delineates diverse genomic landscapes and potential therapeutic targets
Source: Nat Commun. 2022 Jun 15;13:3405. doi: 10.1038/s41467-022-30453-x (PMC9200818; doi:10.1038/s41467-022-30453-x)
Supplement: Supplementary file 1 — Supplementary Information [file 41467_2022_30453_MOESM1_ESM.pdf]

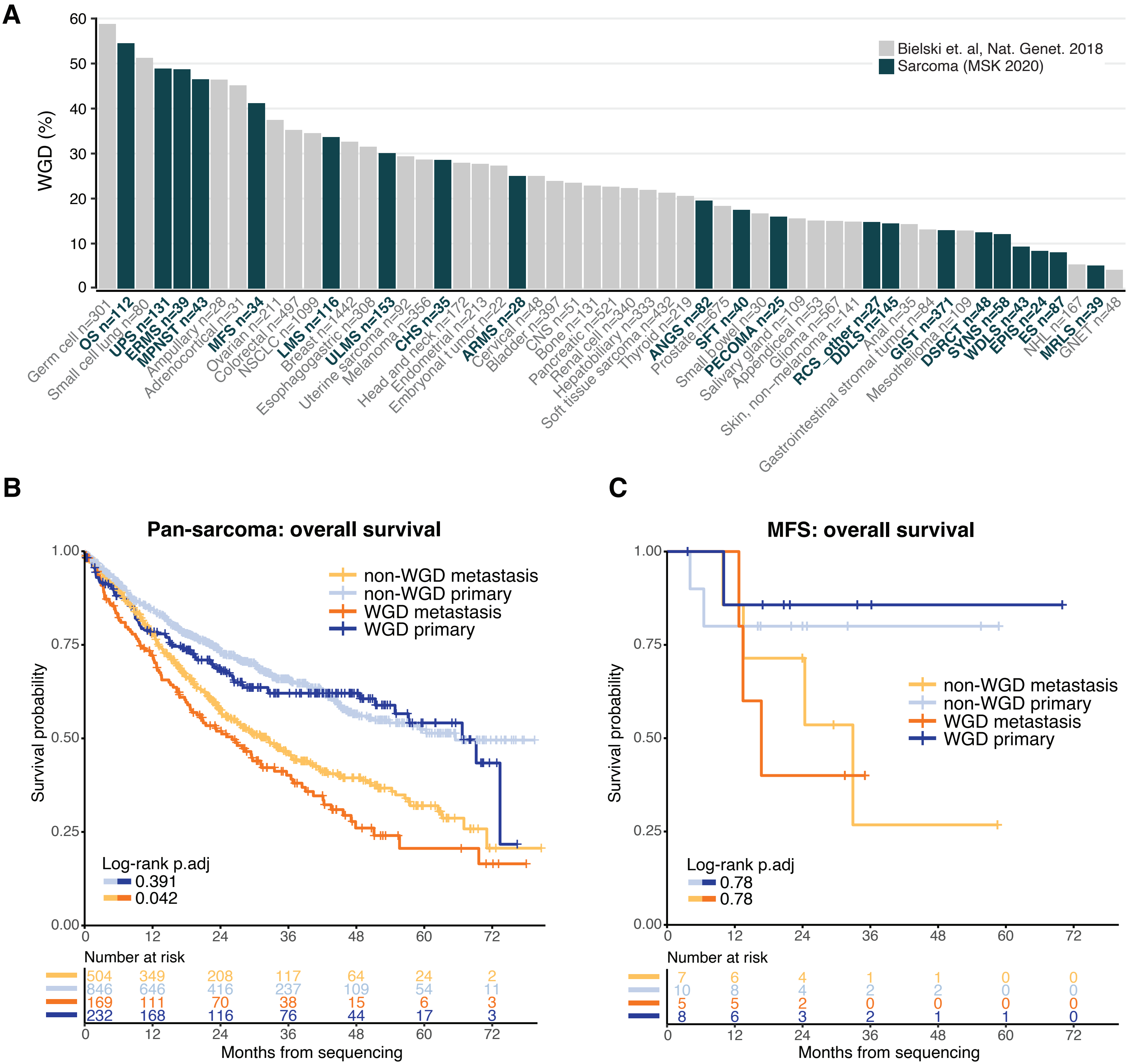

Supplementary Figure 1. Whole genome doubling (WGD) and survival probability. A, Frequency of WGD by subtype (green) compared to other cancers (all available samples) (gray). **B-C**, Overall survival based on WGD status within metastatic and primary tumor cohorts in **B**, all sarcoma subtypes; **C**, MFS.

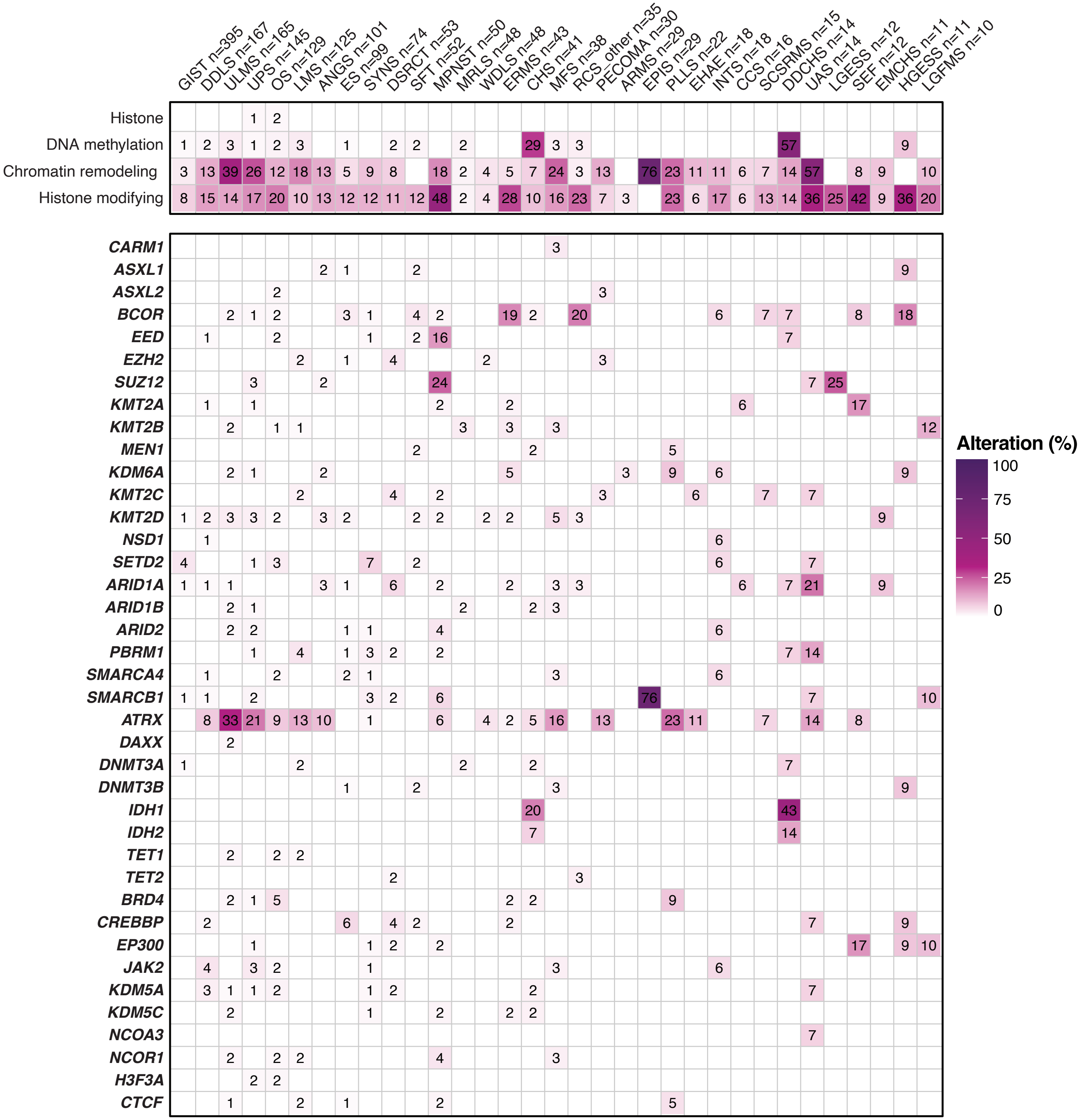

Supplementary Figure 2. Oncogenic epigenetic pathway alterations. Frequency of oncogenic alterations in specific epigenetic pathway genes in each subtype with ≥ 10 samples. Top box, aggregate number of alterations in each gene family/biochemical process.

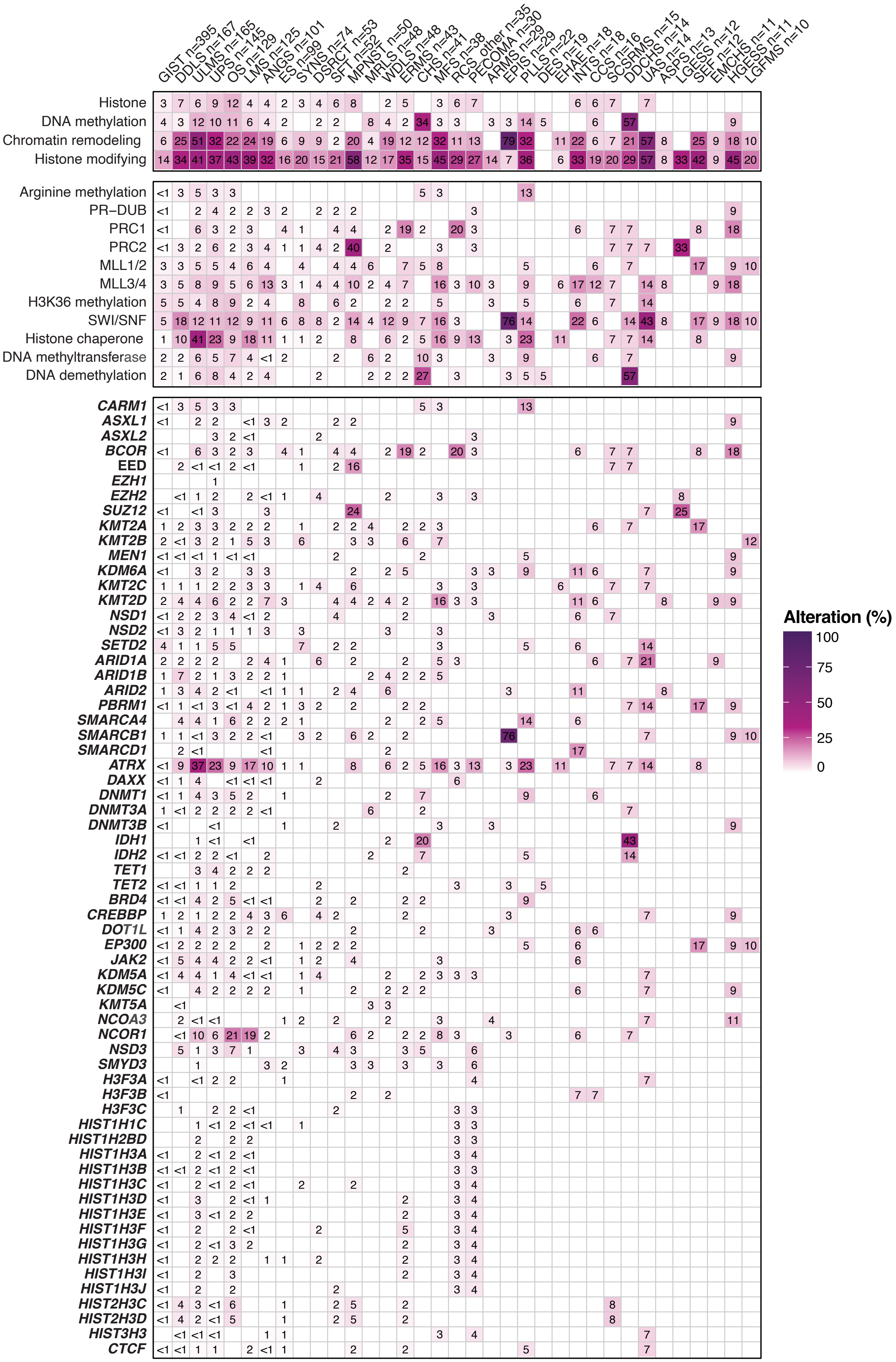

Supplementary Figure 3. All epigenetic pathway alterations, including variants of unknown significance (VUS). Frequency of somatic alterations in epigenetic pathway genes in each subtype with ≥ 10 samples. Top , aggregate number of alterations in each gene family and biochemical process.

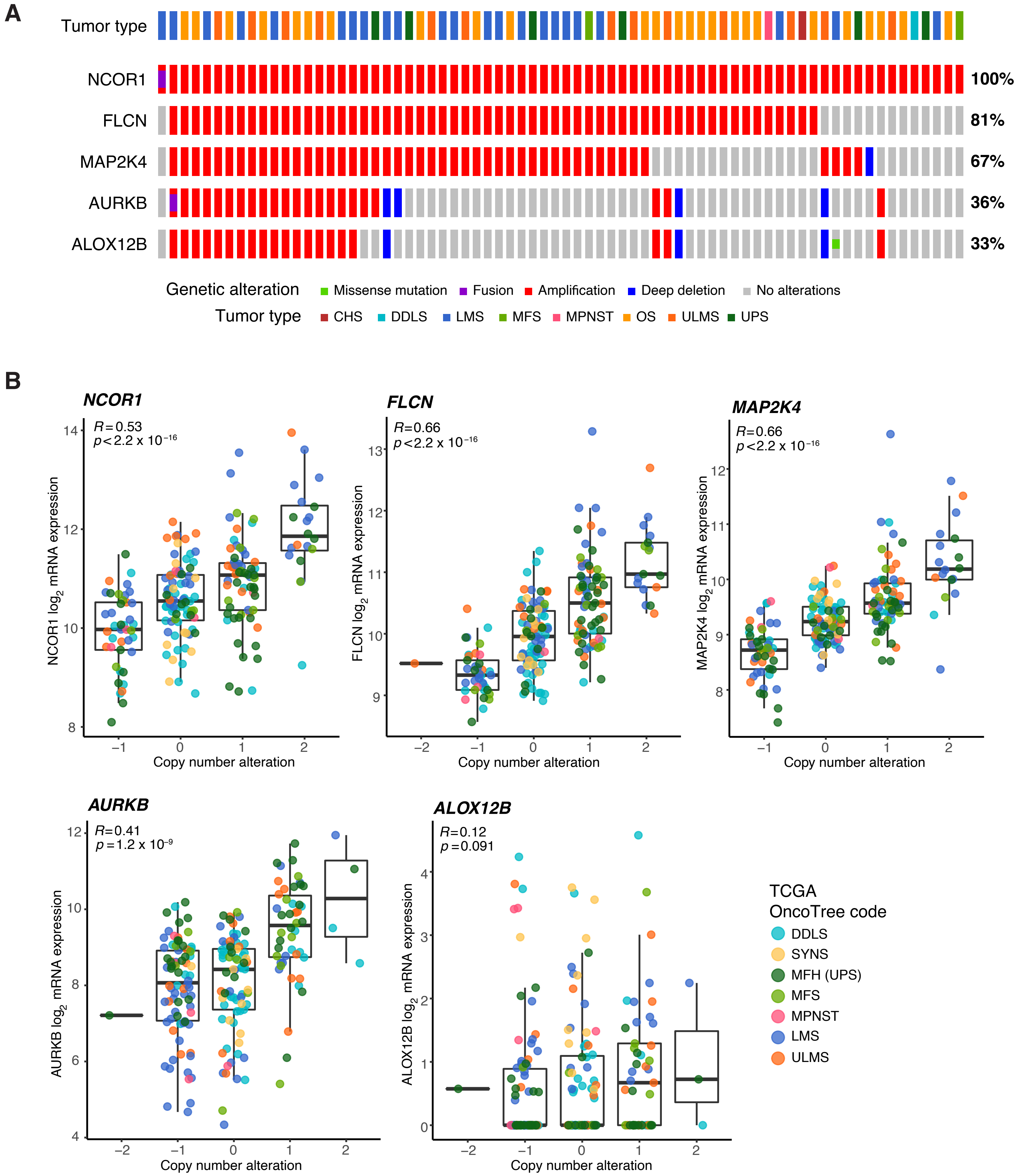

Supplementary Figure 4. Amplification and expression of 17p12-p11.2 genes NCOR1, FLCN, MAP2K4, AURKB, and ALOX12B. A, Oncoprint demonstrating the frequency of co-amplification of other genes at cytoband 17p12-p11.2 in the MSK-IMPACT panel in cases where NCOR1 is amplified. **B**, Correlation of gene expression in the sarcoma TCGA dataset with CNV. Box boundaries indicate 25th and 75th percentiles, interior lines indicate medians, and whiskers indicate 1.5 times the interquartile range. P values calculated by Spearman's rank correlation.

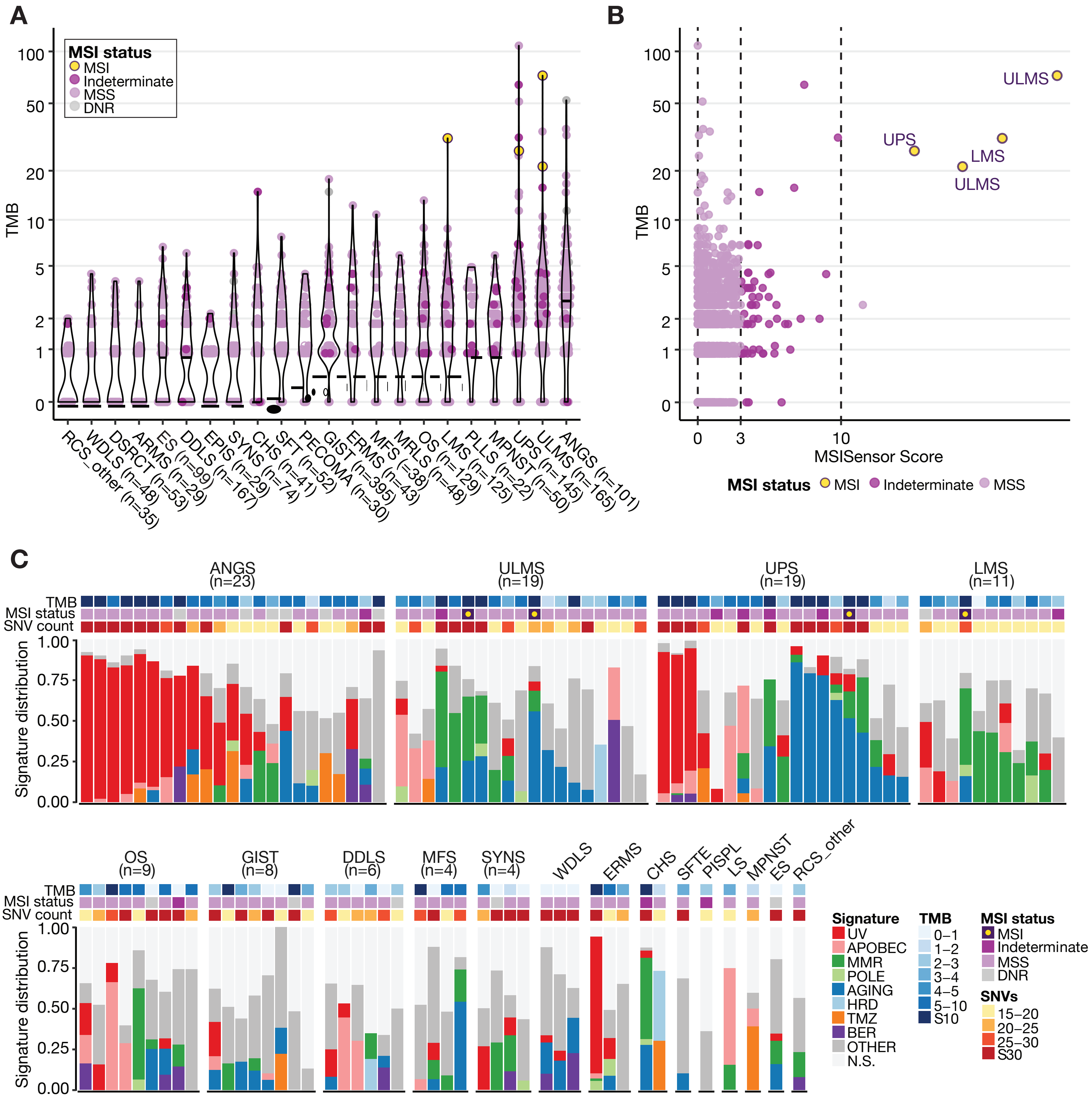

Supplementary Figure 5. Tumor mutation burden (TMB), microsatellite instability (MSI) status, and mutational signatures. A, Distribution of TMB (mutations/Mb) by subtype with sample-level MSI status. **B,** MSISensor score versus TMB for the cohort with sample-level MSI status. **C,** Mutational signature profile of each evaluable sample. Each bar represents the signature distribution of an individual sample, grouped by subtype. TMB, MSI status, and single nucleotide polymorphism (SNV) count are shown above.

Supplementary Table 1. Patient characteristics by subtype.

SAMPLES		AGE		SEX		SAMPLE TYPE	
	**Count**	**Mean ± SD**	**Range**	**Male**	**Female**	**Metastatic**	**Primary**
**TOTAL**	2138	49.3 ± 20.0	0–90	1040	1098	790	1348
**GIST**	395	58.5 ± 14.2	11–90	213	182	123	272
**DDLS**	167	62.1 ± 11.7	29–90	115	52	58	109
**ULMS**	165	57.2 ± 10.3	30–83	0	165	91	74
**UPS**	145	55.6 ± 18.1	3–87	74	71	52	93
**OS**	129	26.1 ± 16.6	8–78	79	50	52	77
**LMS**	125	57.8 ± 14.6	7–83	49	76	71	54
**ANGS**	101	58.6 ± 16.7	15–89	41	60	24	77
**ES**	99	26.9 ± 16.1	2–79	60	39	28	71
**SYNS**	74	40.7 ± 16.0	7–85	36	38	24	50
**DSRCT**	53	23.4 ± 9.4	8–48	48	5	24	29
**SFT**	52	60.2 ± 11.0	39–80	25	27	22	30
**MPNST**	50	43.7 ± 20.2	11–89	30	20	12	38
**MRLS**	48	44.7 ± 13.4	13–67	29	19	16	32
**WDLS**	48	57.8 ± 9.9	34–74	27	21	25	23
**ERMS**	43	15.5 ± 16.8	1–70	20	23	7	36
**CHS**	41	47.9 ± 15.4	16–76	26	15	16	25
**MFS**	38	64.5 ± 12.6	31–80	21	17	13	25
**RCS_other**	35	27.8 ± 18.0	0–70	14	21	3	32
**PECOMA**	30	55.9 ± 15.0	14–76	7	23	15	15
**ARMS**	29	23.5 ± 16.4	1–67	16	13	13	16
**EPIS**	29	38.5 ± 15.2	16–67	14	15	9	20
**PLLS**	22	53.0 ± 18.9	12–87	10	12	8	14

Supplementary Table 2. Number of actionable fusion events in individual genes in each subtype.

Fusion	Total	ANGS	DDLS	ES	GIST	IFS	IMT	LMS	MFS	MRLS	OS	PECOMA	SYNS	UAS	ULMS	UPS	WDLS
**EWSR1-FLI1**	81	0	0	81	0	0	0	0	0	0	0	0	0	0	0	0	0
**ALK**	7	0	0	0	0	0	5	0	0	0	0	0	0	0	2	0	0
**BRAF**	4	1	1	0	0	0	0	0	0	0	0	0	0	0	0	2	0
**NF1**	4	0	0	0	0	0	0	0	0	0	0	1	0	0	1	2	0
**NTRK3**	4	0	0	0	1	2	1	0	0	0	0	0	0	0	0	0	0
**ROS1**	4	0	0	0	0	0	2	0	0	1	0	1	0	0	0	0	0
**NTRK1**	3	1	1	0	0	0	0	0	0	0	0	0	0	0	0	0	1
**TSC2**	3	0	1	0	0	0	0	0	0	0	0	1	1	0	0	0	0
**BRCA2**	2	0	0	0	0	0	0	0	1	0	0	0	0	0	1	0	0
**CDKN2A**	2	1	0	0	1	0	0	0	0	0	0	0	0	0	0	0	0
**NTRK2**	2	0	2	0	0	0	0	0	0	0	0	0	0	0	0	0	0
**PTEN**	2	0	0	0	0	0	0	1	0	0	0	0	0	0	0	1	0
**ATM**	1	0	0	0	1	0	0	0	0	0	0	0	0	0	0	0	0
**CDK12**	1	0	0	0	0	0	0	0	0	0	0	0	0	0	0	1	0
**CHEK2**	1	1	0	0	0	0	0	0	0	0	0	0	0	0	0	0	0
**ESR1**	1	0	0	0	0	0	0	0	0	0	0	0	0	1	0	0	0
**FGFR1**	1	0	1	0	0	0	0	0	0	0	0	0	0	0	0	0	0
**FGFR2**	1	0	0	0	0	0	0	0	0	0	0	0	1	0	0	0	0
**FGFR3**	1	0	1	0	0	0	0	0	0	0	0	0	0	0	0	0	0
**NCOA3**	1	0	0	0	0	0	0	0	0	0	0	0	0	1	0	0	0
**PDGFRA**	1	1	0	0	0	0	0	0	0	0	0	0	0	0	0	0	0
**PTCH1**	1	0	0	0	0	0	0	0	0	0	1	0	0	0	0	0	0
**SUZ12**	1	0	0	0	0	0	0	0	0	0	0	0	0	0	0	1	0

Supplementary Table 3. Biochemical function/complex assignments for epigenetic pathway genes.

**DNA methylation**	
Gene	Group classification
DNMT1	DNA methyltransferase
DNMT3A	DNA methyltransferase
DNMT3B	DNA methyltransferase
IDH1	DNA demethylation
IDH2	DNA demethylation
TET1	DNA demethylation
TET2	DNA demethylation

**Histone modifying**	
Gene	Group classification
ASXL1	PR-DUB
ASXL2	PR-DUB
BCOR	PRC1
BRD4	
CARM1	Arginine methylation
CREBBP	
DOT1L	
EED	PRC2
EP300	
EZH1	PRC2
EZH2	PRC2
JAK2	
KDM5A	
KDM5C	
KDM6A	MLL3/4
KMT2A	MLL1/2
KMT2B	MLL1/2
KMT2C	MLL3/4
KMT2D	MLL3/4
KMT5A	
MEN1	MLL1/2
NCOA3	
NCOR1	
NSD1	H3K36 methylation
NSD2	H3K36 methylation
NSD3	
SETD2	H3K36 methylation
SMYD3	
SUZ12	PRC2

**Chromatin remodeling/histone chaperone**	
Gene	Group classification
ARID1A	SWI/SNF
ARID1B	SWI/SNF
ARID2	SWI/SNF
ATRX	Histone chaperone
DAXX	Histone chaperone
PBRM1	SWI/SNF
SMARCA4	SWI/SNF
SMARCB1	SWI/SNF
SMARCD1	SWI/SNF

**Histone**
Gene
H3F3A
H3F3B
H3F3C
HIST1H1C
HIST1H2BD
HIST1H3A
HIST1H3B
HIST1H3C
HIST1H3D
HIST1H3E
HIST1H3F
HIST1H3G
HIST1H3H
HIST1H3I
HIST1H3J
HIST2H3C
HIST2H3D
HIST3H3

**Other**
CTCF
